# Treatment Modality and Survival in Human Papillomavirus (HPV)-Stratified Early-Stage Oropharyngeal Cancer: A Population-Based Propensity Score-Matched Analysis

**DOI:** 10.7759/cureus.112228

**Published:** 2026-07-07

**Authors:** Adrian L Hoenle, Annekatrin Coordes, Florian Kister, Stephan Lorenz

**Affiliations:** 1 Department of Otorhinolaryngology, Head and Neck Surgery, Klinikum Stuttgart, Stuttgart, DEU; 2 Department of Otorhinolaryngology, Head and Neck Surgery, Brandenburg Medical School, Neuruppin, DEU; 3 Department of Maxillofacial Surgery, Klinikum Saarbrücken, Saarbrücken, DEU; 4 Department of Emergency Medicine, Klinikum Stuttgart, Stuttgart, DEU

**Keywords:** chemoradiotherapy, human papillomavirus (hpv), oropharyngeal cancer, oropharyngeal squamous cell carcinoma, p16, propensity score matching, surgery, surveillance epidemiology and end results (seer), survival, treatment modality

## Abstract

Background: Whether treatment modality effects on survival differ by human papillomavirus (HPV)/p16 status in oropharyngeal squamous cell carcinoma (OPSCC) remains unresolved at the population level.

A propensity score-matched analysis was performed using data from 9,884 patients with Stage I-II OPSCC identified from the Surveillance, Epidemiology, and End Results (SEER) database (2018-2021). Chemoradiotherapy (CRT) vs. surgery (1:1) and CRT vs. radiotherapy (RT) alone (1:2) were compared, stratified by p16 status. A likelihood ratio test formally tested the treatment×HPV interaction on disease-specific survival (DSS).

Results: Surgery was associated with superior overall survival (OS) (hazard ratio (HR) 0.75, 95% CI 0.65-0.87) and DSS (HR 0.59, 0.49-0.72) versus CRT. RT alone was inferior (OS HR 1.55, DSS HR 1.38). The treatment×HPV interaction was non-significant for surgery (DSS p=0.705) and RT alone (DSS p=0.090). In Stage III-IV, no surgical advantage was observed.

Conclusions: Surgical treatment is associated with superior survival in Stage I-II OPSCC irrespective of HPV status. HPV status does not modify the relative effect of treatment modality. RT alone was markedly inferior to CRT in p16-positive Stage II disease.

## Introduction

Human papillomavirus (HPV)-driven oropharyngeal squamous cell carcinoma (OPSCC) has become the predominant subtype in high-income countries, fundamentally altering the epidemiological and prognostic landscape of head and neck oncology [1, 2]. The markedly better outcomes associated with p16-positive disease, with five-year overall survival (OS) exceeding 70% in early-stage patients versus approximately 40% in p16-negative disease, prompted the American Joint Committee on Cancer (AJCC) 8^th^ Edition (2018) to introduce HPV-specific staging criteria [3-5].

This survival divergence has fueled substantial interest in treatment de-escalation for HPV-positive OPSCC, with the hypothesis that less intensive treatment may preserve oncological outcomes while reducing toxicity [6, 7]. Concurrent chemoradiotherapy (CRT) remains the non-surgical standard of care for early and locally advanced OPSCC, but the advent of transoral robotic surgery (TORS) and transoral laser microsurgery has revitalized surgical approaches, particularly for Stage I-II disease [8, 9]. Randomized trials including ORATOR and De-ESCALaTE HPV have compared modalities, yet these studies enrolled predominantly male, p16-positive patients and were not powered to detect differential effects by HPV status [10, 11].

A central unresolved question is whether treatment modality effects on survival differ by p16/HPV status and, specifically, whether HPV status should influence the choice between surgery, CRT, and radiotherapy (RT) alone. Population-based evidence addressing this question with formal interaction testing is lacking. Surveillance, Epidemiology, and End Results (SEER) data from 2018 onwards provide, for the first time, systematically documented p16/HPV status under AJCC 8^th^ Edition criteria, enabling adequately powered population-level analyses [12].

We conducted a propensity score-matched analysis of 9,884 Stage I-II OPSCC patients to address three questions: whether surgical treatment is associated with superior survival compared with CRT in early-stage OPSCC; whether the treatment modality effect is modified by p16/HPV status as tested by a formal treatment×HPV interaction test; and whether the treatment modality effect extends to Stage III-IV disease.

## Materials and methods

Data source and study population

Data were drawn from the SEER program's 17-registry database (November 2024 submission, diagnosis years 2000-2022), accessed using the SEER*Stat software (version 8.4.2; Surveillance Research Program, National Cancer Institute, Bethesda, MD, USA). The SEER program captures approximately 26.5% of the United States population [12]. The analytical cohort was restricted to patients with histologically confirmed OPSCC and known p16/HPV status diagnosed in 2018-2021. Eligible primary sites included the base of the tongue (C01.9), tonsil (C09.0-C09.9), and oropharynx (C10.2, C10.3, C10.8, C10.9); the anterior epiglottis surface (C10.1) was excluded per AJCC 8^th^ Edition anatomical definitions [3]. Patients were excluded if vital status was unknown, age was below 15 years, or survival was recorded as zero months with a concurrent alive status. Given the use of de-identified registry data available in the public domain, ethics committee review was not required.

p16/HPV classification and staging

p16/HPV classification relied on the SEER Schema ID variable introduced with the 2018+ data, which assigns patients to either the HPV-mediated oropharyngeal schema (p16-positive) or the p16-negative oropharyngeal schema, based on p16 immunohistochemistry results serving as a validated proxy for high-risk HPV infection [13,14]. AJCC 8^th^ Edition stage groups were extracted from the Extent of Disease (EOD) 2018 Stage Group field. The primary analysis cohort encompassed Stage I-II patients (n=9,884); an additional 4,081 Stage III-IV patients were included as a secondary analytical cohort.

Treatment classification

Three mutually exclusive treatment groups were defined: CRT (radiation therapy plus chemotherapy), surgery (surgical resection of the primary site, with or without adjuvant treatment), and RT alone (radiation therapy without chemotherapy or surgery). Patients receiving none of these or with undocumented treatment were excluded from survival analyses. CRT served as the reference group for all comparisons, as it represents the established non-surgical standard of care for OPSCC [15,16].

Propensity score matching (PSM)

Separate PSM analyses were conducted for CRT vs surgery (1:1 ratio) and CRT vs RT alone (1:2 ratio). PSM was performed using the MatchIt package in R developed by Ho et al., available from the Comprehensive R Archive Network (CRAN)), with nearest-neighbor matching on estimated propensity scores derived from logistic regression including age, sex, race, diagnosis year, and AJCC stage as covariates [17]. A caliper of 0.2 standard deviations of the logit-transformed propensity score was applied to all models, a widely recommended approach for optimal matching performance. Exact matching was applied for p16/HPV status and AJCC stage within p16-positive patients; for p16-negative patients, exact matching was applied for HPV status only, with stage included as a PSM covariate, owing to small cell sizes in Stage I p16-negative CRT (n=15). Surgery versus RT alone was not compared, as this does not represent a realistic clinical decision in current practice [15,18]. Covariate balance was assessed using standardized mean differences (SMD < 0.10 as a threshold); a Love plot is provided in Appendix A. In p16-negative patients, residual imbalance remained after matching owing to small subgroup sizes; these results should be considered exploratory.

Statistical analysis

Analyses were performed in R (version 4.5.2; R Foundation for Statistical Computing, Vienna, Austria) [19]. Survival was estimated by the Kaplan-Meier method, and differences were assessed by the log-rank test. Multivariable Cox proportional hazards regression estimated OS and disease-specific survival (DSS) hazard ratios (HRs) for the treatment group, adjusting for age, sex, race, and diagnosis year in matched cohorts. The proportional hazards (PH) assumption was assessed using Schoenfeld residuals; where a violation was detected, findings were interpreted accordingly.

The primary analytical focus was the formal treatment×HPV interaction test, implemented as a likelihood ratio test (LRT) comparing Cox models with and without the treatment×HPV interaction term. This test determines whether treatment modality effects on survival differ statistically between p16-positive and p16-negative patients. Interaction tests used DSS as the primary endpoint, as DSS reflects tumor-specific effects without dilution from non-cancer deaths. Stage-specific Cox analyses (Stage I and Stage II separately) were conducted for p16-positive patients; p16-negative patients were analyzed as Stage I-II. I combined due to a limited sample size (n=67 per arm after matching). Secondary analyses used multivariable Cox regression for Stage III-IV disease without PSM, owing to the highly selected nature of surgically treated Stage III-IV patients. All tests were two-sided; significance threshold α = 0.05.

## Results

Cohort characteristics

The treated cohort with known p16 status comprised 17,250 patients: 9,884 with Stage I-II disease (primary analysis) and 4,081 with Stage III-IV disease (secondary analysis). Among Stage I-II patients, 4,922 received CRT, 3,852 underwent surgery, and 1,110 received RT alone. p16-positive disease predominated in all treatment groups (CRT: 98.6%; surgery: 92.5%; RT alone: 91.3%). The RT alone group was the oldest on average (mean age 67.7 years), while CRT patients had a mean age of 62.7 years, and surgery patients were the youngest (61.3 years). Baseline characteristics are presented in Table 1.

**Table 1:** Baseline characteristics by treatment group, Stage I–II oropharyngeal squamous cell carcinoma Descriptive statistics only; no inferential testing performed. CRT: chemoradiotherapy; SD: standard deviation; RT: radiotherapy

Treatment	n	p16+ n (%)	Age mean (in years, SD)	Male n (%)	White n (%)	Stage I n (%)	Stage II n (%)
CRT	4,922	4855 (98.6)	62.7 (9.2)	4364 (88.7)	4486 (91.1)	3023 (61.4)	1899 (38.6)
Surgery	3,852	3564 (92.5)	61.3 (10.0)	3238 (84.1)	3531 (91.7)	3203 (83.2)	649 (16.8)
RT alone	1,110	1013 (91.3)	67.7 (10.4)	918 (82.7)	997 (89.8)	828 (74.6)	282 (25.4)

Propensity score matching

After PSM with caliper restriction, 6,982 patients were matched for the CRT vs surgery comparison (p16+: 6,848; p16−: 134) and 2,982 for CRT vs RT alone (p16+: 2,861; p16−: 121). Within p16-positive strata, stage was perfectly balanced (SMD=0.00 for both Stage I and II), and all other covariates achieved SMD <0.08 for CRT vs surgery and SMD <0.06 for CRT vs RT alone.

Primary analysis: Stage I-II survival

**Figure 1:**
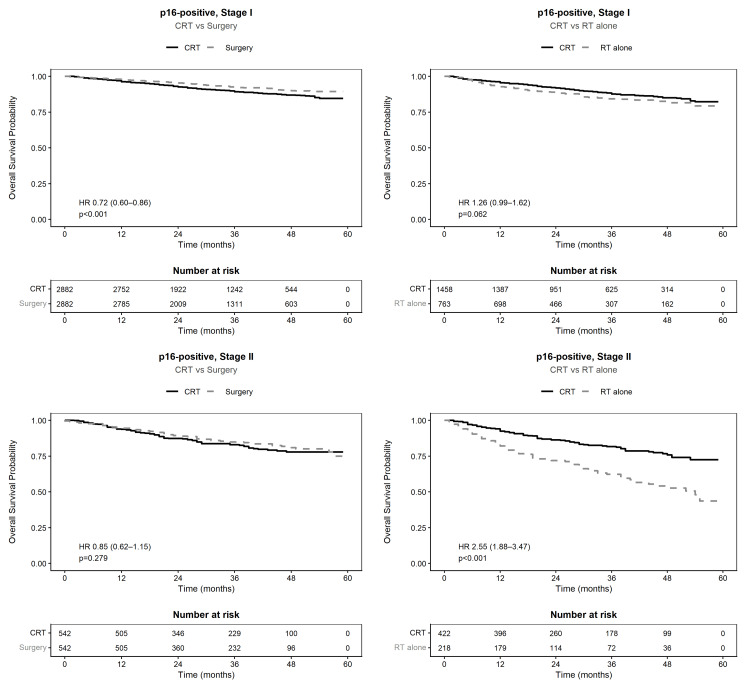
Kaplan-Meier overall survival curves, p16-positive patients by stage Propensity score matched cohort; Left panels: CRT vs. surgery; Right panels: CRT vs RT alone; hazard ratios and p-values from multivariable Cox proportional hazards regression. Number-at-risk tables are shown below each panel. Reference: CRT. CRT: chemoradiotherapy; RT: radiotherapy

**Figure 2:**
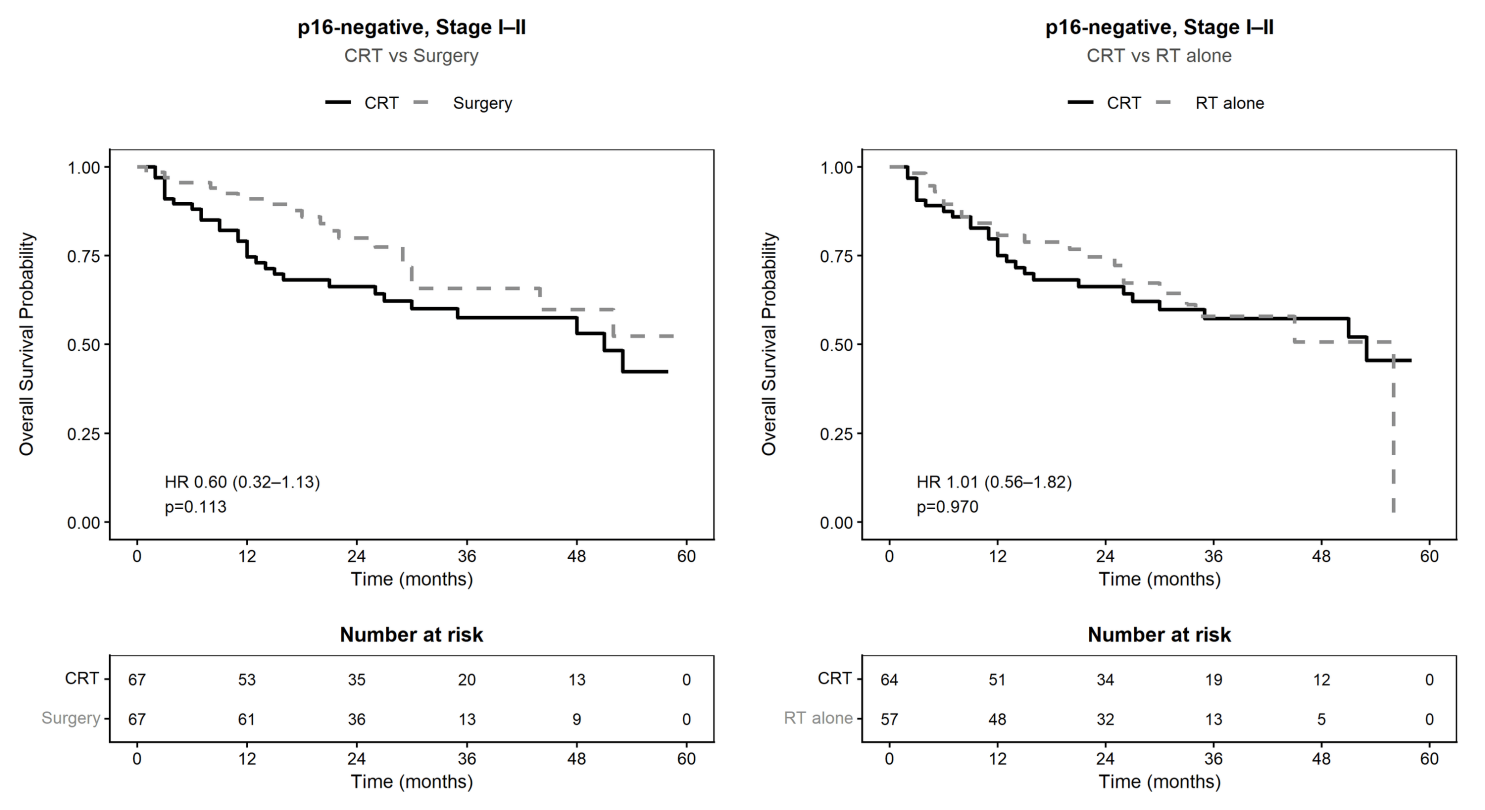
Kaplan-Meier overall survival curves, p16-negative patients (Stage I-II combined) Propensity score-matched cohort: Left: CRT vs surgery; Right: CRT vs RT alone; Hazard ratios and p-values from multivariable Cox proportional hazards regression. Number-at-risk tables are shown below each panel. Reference: CRT. CRT: chemoradiotherapy; RT: radiotherapy

Kaplan-Meier curves are shown in Figures 1, 2. In the combined Stage I-II matched cohort, surgery was associated with significantly superior OS (HR 0.75, 95% CI 0.65-0.87, p<0.001) and DSS (HR 0.59, 95% CI 0.49-0.72, p<0.001) versus CRT. RT alone was associated with significantly inferior OS (HR 1.55, 95% CI 1.29-1.86, p<0.001) and DSS (HR 1.38, 95% CI 1.09-1.75, p=0.007) versus CRT.

Stage-specific analyses in p16-positive patients (Table 2) showed that the surgery advantage was significant in Stage I for both OS (HR 0.72, 95% CI 0.60-0.86, p<0.001) and DSS (HR 0.56, 95% CI 0.44-0.72, p<0.001). In Stage II, the OS difference was non-significant (HR 0.85, p=0.279) while DSS remained significantly better with surgery (HR 0.68, 95% CI 0.47-0.97, p=0.035), suggesting a tumour-specific survival advantage that is attenuated at the OS level by competing causes of death. RT alone was markedly inferior to CRT in Stage II (OS HR 2.55, p<0.001) but only borderline significant in Stage I (OS HR 1.26, p=0.062; Table 3).

**Table 2:** Surgery vs CRT: treatment effect by stage and HPV status HRs and p-values derived from multivariable Cox proportional hazards regression. HR relative to CRT (reference). TX × HPV p: likelihood ratio test for TX× HPV interaction, based on DSS; one p-value per stage stratum applies to both HPV subgroups. — = not shown separately (p16- Stage I–II analyzed as a combined subgroup; Stage III–IV p16- shares the same interaction test as p16+). CRT: chemoradiotherapy; HPV: human papillomavirus; PSM: propensity score matching; Cox: multivariable Cox regression without PSM; OS: overall survival; DSS: disease-specific survival; HR: hazard ratio; CI: confidence interval; TX: treatment

Stage	HPV	OS HR (95% CI)	OS p	DSS HR (95% CI)	DSS p	TX × HPV p (DSS)
I (PSM)	p16+	0.72 (0.60–0.86)	<0.001	0.56 (0.44–0.72)	<0.001	0.541
II (PSM)	p16+	0.85 (0.62–1.15)	0.279	0.68 (0.47–0.97)	0.035	0.685
I–II (PSM)	p16−	0.60 (0.32–1.13)	0.113	0.38 (0.17–0.84)	0.017	—
III–IV (Cox)	p16+	1.10 (0.84–1.42)	0.491	1.18 (0.88–1.57)	0.264	0.15
	p16−	0.89 (0.75–1.06)	0.189	0.96 (0.79–1.16)	0.669	—

**Table 3:** RT alone vs CRT: treatment effect by stage and HPV status HRs and p-values derived from multivariable Cox proportional hazards regression. HR relative to CRT (reference). TX × HPV p: likelihood ratio test for TX × HPV interaction, based on DSS; one p-value per stage stratum applies to both HPV subgroups. — = not shown separately (p16- Stage I–II analyzed as a combined subgroup; Stage III–IV p16- shares the same interaction test as p16+). CRT: chemoradiotherapy; HPV: human papillomavirus; PSM: propensity score matched; Cox: multivariable Cox regression without PSM; OS: overall survival; DSS: disease-specific survival; HR: hazard ratio; CI: confidence interval; TX: treatment

Stage	HPV	OS HR (95% CI)	OS p	DSS HR (95% CI)	DSS p	TX × HPV p (DSS)
I (PSM)	p16+	1.26 (0.99–1.62)	0.062	1.10 (0.79–1.53)	0.565	0.177
II (PSM)	p16+	2.55 (1.88–3.47)	<0.001	2.40 (1.64–3.51)	<0.001	0.076
I–II (PSM)	p16−	1.01 (0.56–1.82)	0.97	0.81 (0.40–1.61)	0.547	—
III–IV (Cox)	p16+	2.28 (1.80–2.89)	<0.001	2.31 (1.76–3.04)	<0.001	0.238
	p16−	1.77 (1.49–2.10)	<0.001	1.88 (1.54–2.29)	<0.001	—

In p16-negative Stage I-II patients, surgery showed a non-significant trend for OS (HR 0.60, 95% CI 0.32-1.13, p=0.113) and a significant DSS advantage (HR 0.38, 95% CI 0.17-0.84, p=0.017). This DSS finding should be interpreted cautiously given the small matched sample size (n=67 per arm) and residual baseline imbalance in the p16-negative cohort. RT alone showed no meaningful difference versus CRT in p16-negative patients (OS HR 1.01, p=0.97).

Treatment×HPV interaction

**Figure 3:**
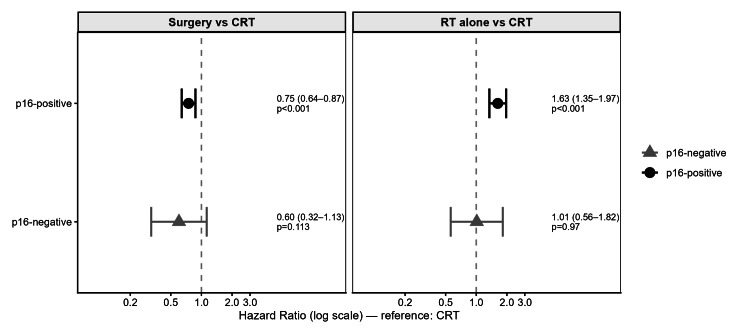
Forest plot of HPV-stratified hazard ratios for OS, Stage I-II Propensity score-matched cohort, reference: CRT; Left panel: Surgery vs CRT; Right panel: RT alone vs CRT; Circles (p16-positive) and triangles (p16-negative) with 95% confidence intervals on a log scale. Interaction p-values from likelihood ratio test (DSS-based): Surgery p=0.705; RT alone p=0.090. HPV: human papillomavirus; CRT: chemoradiotherapy; RT: radiotherapy; DSS: disease-specific survival

The LRT-based treatment×HPV interaction test was non-significant for surgery versus CRT (OS p=0.965; DSS p=0.705), confirming that the survival advantage of surgery over CRT does not differ between p16-positive and p16-negative patients (Table 4, Figure 3). The interaction was also non-significant for RT alone versus CRT (OS p=0.150; DSS p=0.090), indicating that the inferior survival associated with RT alone versus CRT was not statistically different between HPV subgroups after caliper-restricted matching. However, RT alone showed marked inferiority versus CRT specifically in p16-positive Stage II disease (OS HR 2.55, p<0.001; DSS HR 2.40, p<0.001), while no meaningful difference was observed in p16-negative patients (OS HR 1.01, p=0.97). The proportional hazards assumption was verified using Schoenfeld residuals for all primary analysis models; no violation was detected (all global p>0.10).

**Table 4:** HPV-stratified treatment effect on OS and DSS, Stage I–II HRs and p-values derived from multivariable Cox proportional hazards regression. Interaction p-values from likelihood ratio test comparing Cox models with and without the treatment×HPV interaction term, based on DSS. HPV: human papillomavirus; PSM: propensity score matched; CRT: chemoradiotherapy; RT: radiotherapy; OS: overall survival; DSS: disease-specific survival; HR: hazard ratio; CI: confidence interval

Comparison	p16+ OS HR (95% CI)	p16+ OS p	p16+ DSS HR (95% CI)	p16+ DSS p	p16− OS HR (95% CI)	p16− OS p	p16− DSS HR (95% CI)	p16− DSS p	Interaction p (DSS)
Surgery vs CRT	0.75 (0.64–0.87)	<0.001	0.60 (0.48–0.73)	<0.001	0.60 (0.32–1.13)	0.113	0.38 (0.17–0.84)	0.017	0.914
RT alone vs CRT	1.63 (1.35–1.97)	<0.001	1.48 (1.16–1.90)	0.002	1.01 (0.56–1.82)	0.97	0.81 (0.40–1.61)	0.547	0.09

Secondary analysis: Stage III-IV

In Stage III-IV disease, surgery was not associated with improved survival versus CRT in either p16-positive (OS HR 0.97, p=0.626; DSS HR 1.04, p=0.668) or p16-negative patients (OS HR 0.89, p=0.189; DSS HR 0.96, p=0.669). RT alone was markedly inferior to CRT in both subgroups (p16+ OS HR 2.28, p<0.001; p16− OS HR 1.77, p<0.001). The treatment×HPV interaction was non-significant for both comparisons (Surgery p=0.118; RT alone p=0.096). A violation of the proportional hazards assumption was detected for the RT alone versus CRT comparison in Stage III-IV (Schoenfeld global p=0.003 for OS; p=0.001 for DSS), indicating a time-varying treatment effect; Stage III-IV findings for RT alone should therefore be interpreted as average effects over the observation period.

## Discussion

This population-based propensity score-matched analysis of 9,884 Stage I-II OPSCC patients demonstrates that surgical treatment is associated with superior OS and DSS compared with CRT, irrespective of HPV status. The formal treatment×HPV interaction test, the primary methodological contribution of this study, showed no significant interaction for surgery (DSS p=0.705), establishing that HPV status does not modify the relative survival benefit of surgery over CRT at the population level. RT alone showed a non-significant trend toward HPV-dependent interaction (DSS p=0.090), with RT alone markedly inferior to CRT specifically in p16-positive Stage II disease (OS HR 2.55, p<0.001) while showing no meaningful difference in p16-negative patients (OS HR 1.01, 95% CI 0.56-1.82, p=0.97). The marked inferiority of RT alone versus CRT in p16-positive Stage II disease (OS HR 2.55, p<0.001) provides a robust, adequately powered signal against the use of RT alone in HPV-positive OPSCC. In p16-negative patients, the absence of a significant difference reflects limited statistical power (n=67 per arm) rather than equivalence, as the wide confidence interval does not exclude clinically meaningful harm. These findings collectively argue against RT alone as a primary treatment strategy in either HPV subgroup.

The observed surgery advantage (Stage I-II OS HR 0.75, DSS HR 0.59) is consistent with several population-based studies and a recent meta-analysis reporting favourable outcomes with surgical approaches in OPSCC [9,20-22]. Notably, a 2025 meta-analysis of 24 non-randomised studies (n=38,396) found surgery associated with superior OS (HR 0.63) and DFS (HR 0.48) versus non-surgical modalities in HPV-negative OPSCC, while acknowledging substantial confounding bias [20]. No prior meta-analysis has formally tested the treatment×HPV interaction, which is the primary contribution of the present study. It should be noted that a recent meta-analysis by Carvalho et al. (2026), comprising two randomised trials and five retrospective studies, did not demonstrate a significant difference in OS or DFS between CRT and surgery in HPV-positive OPSCC [22]. The divergence between this meta-analysis and the present population-level findings may reflect differences in patient selection, institutional surgical volume, and the absence of formal interaction testing in prior studies. The TORS technique has expanded the feasibility of surgery for T1-T2 oropharyngeal lesions, with demonstrated oncological equivalence or superiority in observational series [9,23]. In clinical practice, multimorbid patients who are less likely to tolerate six weeks of platinum-based CRT, with its attendant nephrotoxicity, mucositis, and nutritional compromise, are often preferentially allocated to surgery, particularly when TORS provides a less systemically demanding treatment option [10,24]. This selection pattern would bias against surgery in OS analyses: if the surgical group contains disproportionately more comorbid patients, competing causes of death would attenuate the observed OS advantage. However, DSS, which counts only OPSCC-attributable deaths and thereby eliminates this competing-cause bias, shows an even stronger surgery advantage (HR 0.59 vs OS HR 0.75). This pattern argues against meaningful confounding of the survival benefit by comorbidity: if multimorbidity were substantially biasing the results, one would expect DSS to be closer to null, not more favourable. The convergent evidence from both endpoints strengthens confidence in the robustness of the surgery advantage observed here.

The randomised evidence, while limited, does not contradict these findings. ORATOR demonstrated comparable quality of life outcomes between TORS-based and radiation-based approaches without significant OS differences [10]. ORATOR2 was halted early due to two grade 5 events in the surgical arm; the subsequently published primary analysis demonstrated excellent outcomes in the RT arm with no recurrences or deaths, while the TOS arm did not meet pre-specified survival thresholds [25]. Both trials were limited by small sample sizes and, in the case of ORATOR2, early termination, which restricts the interpretability of their survival data. De-ESCALaTE HPV showed no difference between cisplatin and cetuximab-based CRT or surgery versus non-surgical approaches [11]. The PATHOS trial, currently reporting adjuvant treatment de-escalation after TORS, is not designed to compare surgery with CRT as primary treatment [26]. Our population-level data, with substantially greater sample sizes than any published randomised trial in this space, provide a complementary perspective: the consistent, interaction-tested signal favouring surgery in Stage I-II OPSCC supports the oncological rationale for surgical approaches where technically feasible.

It is important to acknowledge that RT alone does not represent a curative standard-of-care alternative to CRT or surgery in OPSCC. In current clinical practice, RT alone is reserved for patients deemed unfit for concurrent platinum-based chemotherapy, typically elderly or multimorbid patients, and therefore constitutes a compromise strategy rather than an equivalent treatment option [15,16,18]. It should also be noted that concurrent chemotherapy may represent overtreatment for selected Stage I p16-positive patients, particularly those with T1-2 N0 disease, where de-escalation strategies are being actively investigated in prospective trials [6,10]. The present analysis was not powered to distinguish N0 from N1 subgroups within AJCC 8^th^ Edition Stage I disease, as individual pathological nodal staging is not available for non-surgically treated patients in SEER. The inferior survival observed with RT alone versus CRT in the present analysis must be interpreted in this context: the RT alone group was substantially older (mean age 67.7 vs 62.7 years for CRT) and likely carried a higher comorbidity burden, factors not captured in SEER [8]. Despite this caveat, the consistent and clinically substantial inferiority of RT alone across HPV subgroups, even after propensity score matching for available covariates, is directionally consistent with the established evidence favouring concurrent chemotherapy and supports efforts to add chemotherapy whenever clinically feasible, acknowledging that residual confounding by unmeasured comorbidity cannot be excluded.

The treatment×HPV interaction for RT alone was non-significant after caliper-restricted PSM (DSS p=0.090), indicating that the formal statistical threshold for effect modification was not reached. However, the pattern of findings is clinically informative: RT alone was markedly inferior to CRT specifically in p16-positive Stage II patients (OS HR 2.55, p<0.001; DSS HR 2.40, p<0.001), while showing no meaningful difference in p16-negative patients (OS HR 1.01, p=0.97). This directional pattern is consistent with the biological expectation that p16-positive tumours, while radiosensitive, benefit from the additional radiosensitising effect of concurrent chemotherapy when treated non-surgically [4,16,27]. The absence of formal interaction significance likely reflects the limited power of the p16-negative comparison (n=67 per arm after matching) rather than true equivalence of the RT-alone effect across HPV subgroups, as the wide confidence interval in p16-negative patients (OS HR 1.01, 95% CI 0.56-1.82) does not exclude clinically meaningful harm. These findings collectively argue against RT alone as a primary treatment strategy in either HPV subgroup. Methodologically, a non-significant treatment×HPV interaction (DSS p=0.090) implies that the inferior survival associated with RT alone versus CRT cannot be shown to differ between HPV subgroups; the robust signal in p16-positive patients therefore extends, by inference, to p16-negative patients as well. Furthermore, since the only systematic difference between RT alone and CRT is the addition of concurrent chemotherapy, these data implicate concurrent chemotherapy, through direct cytotoxicity and radiosensitisation, as the critical determinant of survival benefit in non-surgically treated OPSCC, irrespective of HPV status [4,15,16]. It should be noted that SEER does not distinguish between platinum-based and cetuximab-based chemotherapy regimens; the observed benefit may reflect cisplatin-specific radiosensitisation and should be interpreted in that context [7,11].

The absence of a surgery advantage in Stage III-IV disease is consistent with current guideline recommendations and biological plausibility: locally advanced OPSCC requires the systemic radiosensitising effect of concurrent chemotherapy, and surgical resection of T3-T4 lesions carries substantially greater morbidity without established survival benefit over definitive CRT [15,16]. The proportional hazards violation detected for RT alone in Stage III-IV (Schoenfeld global p<0.01) further cautions against simple interpretation of Stage III-IV RT alone hazard ratios; the time-varying nature of the effect may reflect early treatment failure followed by disease stabilisation in a subset of patients.

The study period of 2018-2021 predates the introduction of AJCC Version 9 (effective January 2026). These findings constitute a definitive population-level characterisation of the AJCC 8^th^ Edition era and provide a benchmark for future cohorts staged under AJCC Version 9.

Several limitations warrant acknowledgement. As a registry-based analysis, SEER lacks smoking history, comorbidity, performance status, and institutional volume data, precluding complete confounding adjustment [28,29]. p16 immunohistochemistry is a validated but imperfect surrogate for HPV status [13,14]. PSM balance in p16-negative patients was limited by small sample sizes (n=67 per arm); these results are considered exploratory. Registry-based staging in SEER reflects clinical rather than pathological nodal staging, and the two approaches show considerable discordance under AJCC 8^th^ Edition criteria, a limitation particularly relevant for non-surgically managed patients in whom pathological staging is unavailable [3,30]. Additional limitations include the absence of data on radiation technique, including intensity-modulated radiation therapy, volumetric modulated arc therapy, three-dimensional conformal radiation therapy, or particle therapy, radiation dose, surgical margin status, postoperative adjuvant therapy decisions including trimodality treatment, primary disease subsite within the oropharynx, treatment-related toxicity and quality-of-life outcomes, and perioperative morbidity and mortality.

These factors may independently influence survival outcomes and treatment selection, and their absence represents an inherent constraint of population-based registry analyses.

## Conclusions

In this population-based observational analysis of Stage I-II OPSCC, surgical treatment was associated with superior OS and DSS compared with CRT, with a survival advantage that was not modified by HPV/p16 status. These findings suggest that surgical approaches may be considered in early-stage OPSCC independent of p16 status, and that p16 positivity alone should not be used as the sole criterion to favour CRT over surgery in observational data. RT alone was markedly inferior to CRT in p16-positive Stage II disease, and the absence of a significant difference in p16-negative patients reflects limited statistical power rather than equivalence. These findings collectively argue against RT alone as a primary treatment strategy in either HPV subgroup, noting that RT alone does not represent a curative-intent standard in current OPSCC practice and is typically reserved for patients unfit for chemotherapy. In Stage III-IV disease, no survival advantage for surgery over CRT was demonstrated in either HPV subgroup, consistent with current guidelines. Prospective randomised data, with mandatory HPV stratification and formal interaction testing, are needed to confirm these population-level observations.

## Figures and Tables

**Figure 4:**
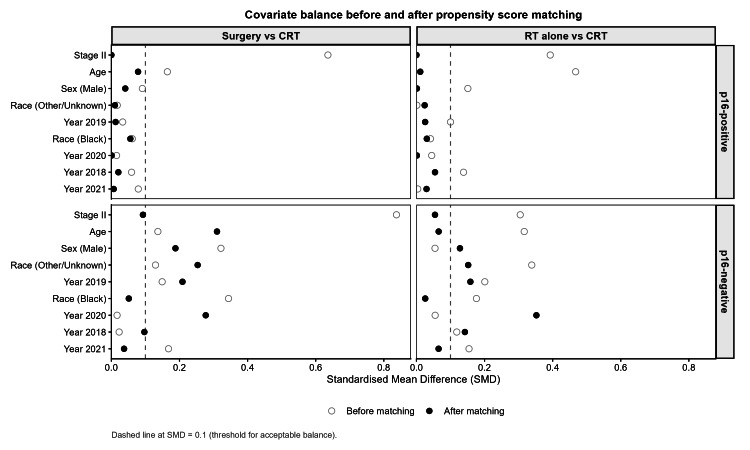
Love plot: covariate balance before and after propensity score matching Stratified by comparison (Surgery vs CRT; RT alone vs CRT) and HPV status (p16-positive; p16-negative). Dashed line at SMD = 0.10 indicates the threshold for acceptable covariate balance. All covariates in the p16-positive strata achieved SMD <0.10 after matching. Residual imbalance in the p16-negative strata reflects limited subgroup sample sizes; p16-negative results are considered exploratory. SMD: standardised mean difference; HPV: human papillomavirus; CRT: chemoradiotherapy; RT: radiotherapy

## Appendices

 Appendix A
